# Dusting versus fragmentation for renal stones during flexible ureteroscopy

**DOI:** 10.1080/2090598X.2019.1601002

**Published:** 2019-04-26

**Authors:** Ahmed R. El-Nahas, Shabir Almousawi, Yaqoub Alqattan, Imad M. Alqadri, Tariq F. Al-Shaiji, Abdullatif Al-Terki

**Affiliations:** a Urology Unit, Surgery Department, Al-Amiri Hospital, Kuwait City, Kuwait; b Urology and Nephrology Center, Mansoura University, Mansoura, Egypt

**Keywords:** Flexible ureteroscopy, renal calculi, stones, Dusting, laser

## Abstract

**Objectives**: To compare stone dusting and spontaneous passage vs fragmentation and active fragment retrieval during flexible ureteroscopy (fURS) for renal calculi.

**Patients and methods**: The study included patients who underwent fURS and holmium laser lithotripsy for renal calculi from January 2015 to March 2017. Dusting was done using low energy and high frequency (0.3–0.5 J and 15–20 Hz, respectively), and fragmentation was done with higher energy and lower frequency (1–1.2 J and 6–10 Hz, respectively) and then stone fragments were extracted using a basket. The stone-free rate (SFR) was evaluated after 2 months with non-contrast computed tomography. Operative time, complication rate, SFR, and the need for secondary procedures were compared.

**Results**: The study included 107 consecutive patients, with a mean (SD) age of 49 (13) years. Dusting was performed in 51 patients and fragmentation in 56. The patients’ demographics, laboratory tests, preoperative stents, stone and renal characteristics were comparable for both groups. Operative time was significantly shorter for dusting than fragmentation (76 vs 91 min, *P* = 0.009). Complication rates were comparable between the groups (7.8% for dusting and 8.9% for fragmentation, *P* = 0.840). The mean hospital stay was comparable for both groups (*P* = 0.686). The SFR was significantly better in fragmentation group (78.6%) compared with the dusting group (58.6%, *P* = 0.035). The need for a secondary procedure was 33.3% in the dusting group and 23.3% in fragmentation group (*P* = 0.244).

**Conclusions**: During fURS for renal stones, the dusting technique had a significantly shorter operation time, whilst the fragmentation technique led to a significantly better SFR. Both techniques have comparable safety, hospital stay, and requirement for secondary procedures.

**Abbreviations:** fURS: flexible ureteroscopy/ureteroscope; ICU: intensive care unit; KUB: plain abdominal radiograph of the kidney, ureter and bladder; NCCT: non-contrast CT; SFR: stone-free rate; SWL: shockwave lithotripsy; UAS: ureteric access sheath

## Introduction

Flexible ureteroscopy (fURS) is currently the preferred treatment option for most uncomplicated renal calculi [1]. This has resulted from the marked improvement in fURS designs, laser lithotripsy machines and techniques, as well as working instruments [2]. It has been shown to be more effective than extracorporeal shockwave lithotripsy (SWL) for small calculi (<20 mm) [3,4], but can still be an effective treatment for stones> 20 mm [5].

The holmium:yttrium-aluminium-garnet (YAG) laser has become the preferred lithotripter device because of its high efficacy and the availability of small-diameter (200 µm) flexible laser fibres, which can pass through the fURS and reach any site in the calyceal system [2]. Holmium laser lithotripters allow the urologist to control laser settings (energy and frequency) to adjust the power that is delivered at the tip of the laser fibre [6]. Low energy (0.2–0.5 J), high frequency (15–40 Hz) lithotripsy results in tiny fragment sizes that can pass spontaneously and this technique has been termed ‘dusting’. On the other hand, higher energy levels (1–1.2 J) with lower frequencies (6–10 Hz) results in fragments that require active retrieval with baskets and this technique has been termed ‘fragmentation’ [7,8].

The widespread use of holmium laser lithotripsy has created debates about the best laser lithotripter settings [9,10]. A few studies have compared fragmentation and active retrieval with dusting and spontaneous passage for renal stones [11,12], and the laser settings were not mentioned in one of them [11]. The present study was conducted to compare two techniques of holmium laser lithotripsy (dusting and spontaneous passage with fragmentation and active basket extraction of stone fragments) during fURS for renal stones.

## Patients and methods

Patients who underwent fURS for renal calculi from January 2015 to March 2017 were retrospectively reviewed. The study included patients who underwent laser disintegration of their stones. Patients who did not complete the procedure due to URS or laser malfunction were excluded. Preoperative evaluation included: urine analysis and culture, serum creatinine, and non-contrast CT (NCCT). All patients received prophylactic i.v. third-generation cephalosporin. Patients with infected urine cultures received culture-specific antibiotics for 5 days before any intervention.

### Technique of fURS

Under general anaesthesia and in lithotomy position, a guidewire (Sensor™; Boston Scientific, Marlborough, MA, USA) was placed via cystoscopy under fluoroscopic guidance into the renal pelvis. Then a dual-lumen catheter (Cook Urological Inc., Bloomington, IN, USA) was used to place a second guidewire. A fURS (FlexX2^TM^ or FlexXC^TM^, Karl Storz Endoskope, Tuttlingen, Germany) was introduced over the second guidewire or through a ureteric access sheath (UAS) of 11/13 F (Navigator™; Boston Scientific).

A holmium laser (CALCULASE®; Karl Storz Endoskope) or (VersaPulse PowerSuit 60 W; Lumenis) was used for lithotripsy. According to the judgement of the attending surgeon, stones were either dusted or fragmented. Dusting was done using low energy and high frequency (0.3–0.5 J and 15–20 Hz), and the tip of the laser fibre was moved over the stone surface (painting movement). Whilst for fragmentation higher energy and lower frequency (1–1.2 J and 6–10 Hz) was used and the stone was disintegrated into fragments that were extracted using a nitinol basket (Zero-Tip™; Cook Urological Inc.). Patients who underwent dusting represent ‘Group D’ and those who underwent fragmentation comprised ‘Group F’. After completing stone dusting or fragmentation, the ureter was inspected during withdrawal of the fURS for any injury caused by the UAS. A double pigtail ureteric stent was placed at the end of the procedure in patients who needed a UAS and if intraoperative ureteric injury was inflicted. A ureteric catheter was placed in the other patients for 24–48 h. Operative time was measured from cystoscope placement until fixation of the urethral catheter. Hospital stay included days in hospital for the first and for the second session of fURS if needed.

### Evaluation

Intraoperative complications were graded according to the Traxer and Thomas grading of endoscopic ureteric injury [13]. A plain abdominal radiograph of the kidney, ureter and bladder (KUB) was taken after 1 day to confirm proper placement of the ureteric stent or catheter. Postoperative complications were classified according to the modified Clavien classification [14]. Another KUB was performed after 3–4 weeks. The ureteric stents were removed under local anaesthesia for patients who had no residual fragments, whilst they were removed in the operating theatre for those who had residual stones and a second session of fURS was performed to retrieve these residuals. The stone-free rate (SFR) was evaluated after 2 months with NCCT. Patients with residual stones were followed-up every 3 months.

### Statistical analysis

Statistical analysis was performed using the Statistical Package for the Social Sciences, version 20 (SPSS®; SPSS Inc., IBM Corp., Armonk, NY, USA). Operative time, complication rate, SFR and the need for secondary procedures were compared between groups D and F. The chi-squared test was used when appropriate to compare categorical data, whilst the *t*-test was used for continuous data comparison between groups. A *P* < 0.05 was considered to indicate statistical significance.

## Results

Of 114 patients who were scheduled for fURS for renal stones, three patients were excluded due to failure to reach the stones and they underwent minimally invasive percutaneous nephrolithotomy (mini-PERC). Another four patients were excluded because the procedure was not completed due to laser malfunction in two and fURS malfunction in the other two. They underwent SWL for their residual stones.

The study thus included 107 consecutive patients, with mean (SD) age of 49 (13) years. Group D included 51 patients and Group F included 56. Patients’ demographics, laboratory tests, preoperative stents, stone and renal characteristics were comparable for both groups (Table 1). In two-thirds of the patients in each group ureteric stents were already present before fURS. In another three patients in Group F, ureteric stents were placed because the UAS could not be advanced through the ureter, and then the fURS was performed 2 weeks later.

**Table 1.** Patients’ demographics, stone and renal characteristics.

Variable	Group D (Dusting)	Group F (Fragmentation)	*P*
***N* (%):**			
Patients			0.962*
Male	28 (55)	31 (55.4)	
Female	23 (45)	25 (44.6)	
ASA Score			0.452*
1	31 (60.8)	30 (53.6)	
2	20 (39.2)	26 (46.4)	
Side			0.168*
Right	17 (33.3)	26 (46.4)	
Left	34 (66.7)	30 (53.6)	
UTI (positive culture)	12 (23.5)	15 (26.8)	0.699*
Recurrent stone disease	19 (37.3)	18 (32.1)	0.579*
Stone number			0.789*
Single	26 (51)	30 (53.6)	
Multiple	25 (49)	26 (46.4)	
Stone site			0.168*
Renal pelvis	11 (21.6)	12 (29.4)	
Calyceal	25 (49)	20 (35.7)	
Multiple sites	15 (29.4)	24 (42.9)	
Preoperative stent	34 (66.7)	38 (67.9)	0.896
**Mean (SD):**			
Age, years	49.2 (13.2)	50.5 (13.4)	0.618^#^
Creatinine, µmol/L	86.7 (9.1)	82.3 (18)	0.212^#^
Stone length, mm	14.5 (5.5)	14.4 (6.9)	0.945^#^

*chi-squared test; **^#^***t*-test.

Intraoperative data and postoperative outcomes are presented in Table 2. Operative time was significantly shorter for Group D than for Group F (76 vs 91 min, *P* = 0.009). The overall complication rates were comparable between the groups (*P* = 0.840). There were two intraoperative complications in Group F, in the form of ureteric perforation (Grade 3 injury). The two injuries were seen during removal of the UAS and were managed by fixation of ureteric stents for 4 weeks. Postoperative fever (> 38°C) occurred in three patients in each group and they were successfully managed by i.v. antibiotics and antipyretics. Gross haematuria was encountered in one patient in Group F and he required 1 unit of blood transfusion. One patient in Group D developed septic shock that required admission to the intensive care unit (ICU) and was successfully treated with i.v. fluids, cardiac inotropics, and a culture-specific antibiotic.

**Table 2.** Intraoperative and postoperative data.

Variable	Group D (Dusting)	Group F (Fragmentation)	*P*
***N* (%):**			
Access			<0.001*
Guidewire	22 (43)	7 (12.5)	
UAS	29 (56)	49 (87.5)	
Complications:	4 (7.8)	5 (8.9)	0.840*
Intraoperative	0 (0)	2 (3.6)	
Postoperative	4 (7.8)	4 (7.1)	
Grade I	3	3	
Grade II	0	1	
Grade IVa	1	0	
Second session of fURS	12 (23.5)	9 (16)	0.332*
Results of fURS:			0.035*
Stone free	30 (58.8)	44 (78.6)	
Insignificant residuals (<4 mm)	14 (27.5)	5 (8.9)	
Residuals (≥4 mm)	7 (13.7)	7 (12.5)	
**Mean (SD):**			
Operative time, min	75.8 (29.6)	91.2 (30.2)	0.009^#^
Hospital stay, days	2.5 (1.3)	2.5 (0.9)	0.686^#^

*chi-squared test; **^#^***t*-test.

The mean hospital stay was comparable for both groups (*P* = 0.686). A second session of fURS was required in 23.5% vs 16% of patients in Group D and Group F, respectively (*P* = 0.332). The SFR after 2 months of fURS was significantly better in Group F (78.6%) compared with Group D (58.6%, *P* = 0.035). The follow-up for patients with residual stones during a median (range) period of 6 (3–12) months is summarised in Table 3. One of the two patients who had intraoperative ureteric perforation developed stricture at the site of injury, which was treated with laser endoureterotomy.

**Table 3.** Follow-up for patients with residual stones.

Variable	Group D (Dusting)	Group F (Fragmentation)
Number of patients	21	12
Treatment during follow-up, *n*	7	5
SWL	2	2
Semi-rigid URS (slipped to the ureter)	2	1
fURS (growth of residuals)	3	1
Spontaneous passage of residuals, *n*	5	1
Same size of residuals	6	5
Lost to follow-up	3	2

## Discussion

Treatment goals for renal stones are a stone-free status using a minimally invasive procedure with the lowest rate of complications. Using laser lithotripsy through fURS enables urologists to achieve most of these goals because of the effectiveness of laser lithotripsy for all stone compositions [8]. Two techniques of laser lithotripsy have recently been studied. The first is fragmenting the stone then basket retrieval of the fragments [7,8] and the second is stone dusting followed by spontaneous passage [11,12].

Stone fragmentation and basket extraction is suggested to have the advantage of a better SFR [15]. In the present study, the SFR of Group F (78.6%) was significantly better than Group D (58.6%). In a randomised multicentre study conducted by Humphrey et al. [12], similar results were reported for SFR (74.3% for fragmentation and basketting vs 58.2% for dusting, *P* = 0.004). They defined SFR as no residual fragments of any size on KUB and renal ultrasonography. Lee et al [11] reported a better SFR (89% for fragmentation vs 86.8% for dusting) than our present study because they considered residual fragments <3 mm as stone free, whereas in our study we defined stone free as no residual fragments. From the patients’ perspective, being free of any stone residuals after a single intervention is more attractive than the anxiety of waiting for weeks to pass the stone fragments, even if they are very small. Moreover, passage of stone fragments is associated with more emergency department visits and renal colic [16]. Several recent modifications in laser machines have included very high frequency (50–80 Hz) [17] or long pulse duration [18] to improve the SFR of the dusting technique. However, these modifications are only available in high-power laser machines (120 W), which are more expensive than 20- or 30-W laser machines.

The superior SFR of the fragmentation technique was associated with a longer operative time. In the present study, the operative time was significantly longer in Group F (91 vs 76 min). In the Humphrey et al. [12] study, the operative time was 37.7 min longer in their fragmentation group (*P*= < 0.001). Longer operative time has been reported to increase the cost of the operation by $29–80/min (American dollars) [19]. It can make operative lists busier and may decrease the number of patients for each list. Other drawbacks of the fragmentation technique are the need for a UAS and the requirement of nitinol baskets to extract the stone fragments [15]. The cost of these instruments will add to the already higher cost of the technique. The UAS facilitates passing the fURS many times, protects the ureter from the wear and tear of multiple instrument passages, whilst minimising intrarenal pressures [20]. However, passage of the UAS was not successful in three patients without a preoperative stent in the present study, which led to the placement of a ureteric stent and postponing of the fURS to allow for passive dilatation of the ureter. Moreover, a UAS may cause ureteric injury [13]. In the present study, the only two operative complications were recorded in Group F, in the form of ureteric perforation (Grade 3 injury according to Traxer and Thomas grading [13]).

The main advantage of the stone dusting technique is the ability to complete the procedure by a single pass of the fURS that can be done over a guidewire. Therefore, the need for a UAS was significantly lower in Group D in the present study (*P* < 0.001). This translated in to the avoidance of intraoperative complications and a significantly shorter operative time in Group D. On the other hand, working without a UAS can lead to an increase in intra-pelvic pressure. This was the most probable reason for development of septic shock requiring ICU admission in one patient in Group D in the present study. Finally, the SFR of the dusting technique was significantly lower than fragmentation because of the inability to be sure that the stone is completely dusted to tiny fragments that are small enough to pass spontaneously without complications.

In the present study, the safety of both techniques was comparable as there was no statistical difference in complication rates (8.9% for fragmentation vs 7.8% for dusting, *P* = 0.840). Lee et al. [11] have reported comparable complications rates (11.8% for fragmentation vs 9.9% for dusting). Similarly, Humphrey et al. [12] reported no significant difference in complications of their dusting and fragmentation groups. Chew et al. [16] studied the natural history of residual fragments after fURS. They found that 29% of patients required a secondary intervention but there was no significant difference in the need for intervention between the dusting and fragmentation techniques. We observed similar results amongst our present patients (33% in the dusting group and 23% in the fragmentation group, *P* = 0.244). The total cost was not calculated in the present study, but it seems that the first session of fURS and dusting would be less costly because of the shorter operation time and lesser need of auxiliary instruments, such as UAS and nitinol baskets. It has to be mentioned that secondary interventions for events caused by residual stones (such as SWL, semi-rigid URS and fURS) also need to be considered.

Limitations of the present study include its retrospective nature and relatively small sample size. The retrospective study design may cause selection bias for choosing the technique of laser lithotripsy, use of UAS, and postoperative stent placement. The small sample size may mask the statistical significance of important differences, such as complication rates and the need for secondary procedures. However, our results are in concordance with a prospective randomised multicentre trial comparing dusting and fragmentation [12]. There is a need for more sufficiently powered randomised trials. Meanwhile, and in the absence of high-level evidence-based recommendations, the urologist has to counsel the patients about the advantages and disadvantages of each laser lithotripsy technique.

## Conclusion

For fURS for renal stones, the dusting technique had a significantly shorter operation time, whilst the fragmentation technique had a significantly better SFR. Both techniques have comparable safety, hospital stay and requirement for secondary procedures.
